# The *Rg1 *allele as a valuable tool for genetic transformation of the tomato 'Micro-Tom' model system

**DOI:** 10.1186/1746-4811-6-23

**Published:** 2010-10-07

**Authors:** Lilian E Pino, Simone Lombardi-Crestana, Mariana S Azevedo, Danielle C Scotton, Lucélia Borgo, Vera Quecini, Antonio Figueira, Lázaro EP Peres

**Affiliations:** 1 Department of Biological Sciences (LCB), Escola Superior de Agricultura "Luiz de Queiroz" (ESALQ), Universidade de São Paulo (USP), Av. Pádua Dias, 11, CP 09, Piracicaba, SP, 13418-900, Brazil; 2 Centro de Energia Nuclear na Agricultura (CENA), USP, Av. Centenário, 303, Piracicaba, SP, 13400-970, Brazil; 3 CNPUV, EMBRAPA, Rua Livramento, 515, CP 130, Bento Gonçalves, RS, 95700-000, Brazil

## Abstract

**Background:**

The cultivar Micro-Tom (MT) is regarded as a model system for tomato genetics due to its short life cycle and miniature size. However, efforts to improve tomato genetic transformation have led to protocols dependent on the costly hormone zeatin, combined with an excessive number of steps.

**Results:**

Here we report the development of a MT near-isogenic genotype harboring the allele *Rg1 *(MT-*Rg1*), which greatly improves tomato *in vitro *regeneration. Regeneration was further improved in MT by including a two-day incubation of cotyledonary explants onto medium containing 0.4 μM 1-naphthaleneacetic acid (NAA) before cytokinin treatment. Both strategies allowed the use of 5 μM 6-benzylaminopurine (BAP), a cytokinin 100 times less expensive than zeatin. The use of MT-*Rg1 *and NAA pre-incubation, followed by BAP regeneration, resulted in high transformation frequencies (near 40%), in a shorter protocol with fewer steps, spanning approximately 40 days from *Agrobacterium *infection to transgenic plant acclimatization.

**Conclusions:**

The genetic resource and the protocol presented here represent invaluable tools for routine gene expression manipulation and high throughput functional genomics by insertional mutagenesis in tomato.

## Background

The miniature tomato (*Solanum lycopersicum *L) cultivar Micro-Tom (MT) is considered a model system for tomato genetics [1] and functional genomics [2,3]. As a model system, MT displays traits comparable to *Arabidopsis*, such as small size and short life cycle, suitable for large scale mutagenesis [1,4,5] and transgenic plant production [1,6]. Further, tomato is the model of choice to study fleshy fruit development, whose knowledge can be extended to other important crops [7].

*Agrobacterium*-mediated transformation of tomato has been explored since the 1980 s, when McCormick and collaborators performed transformation assays of leaf discs from various cultivars [8]. Since then, several reports on genetic transformation of tomato have been published. Transformation efficiencies ranging from 10 to 33% were obtained for several cultivars [8-11], while specifically for MT, rates from 20 to 56% were reported [2,3,12,13]. Many parameters affecting transformation efficiency have been tested, including *in vitro *regeneration ability, which is basically determined by the explant genotype.

The wild species *Solanum peruvianum *is known to exhibit high *in vitro *shoot regeneration ability [14]. At least part of this capacity is due to the presence of the *Rg1 *allele [15], which was mapped to chromosome 3, close to the *yellow fresh *(*r*) locus [16]. The recessive *r *allele, also present in the green fruited species *S. peruvianum*, confers yellow color to fruits in *S. lycopersicum *background, and it can be used as a morphological marker for the presence of *Rg1*. Different from induced mutations conferring altered hormone responses and/or high regeneration capacity, *Rg1 *is a natural genetic variation introgressed from a tomato wild relative, and as such it tends to behave like an adaptive trait, minimizing secondary deleterious effects. Further, the high regeneration capacity of *Rg1 *allows reduced exposure to exogenous hormone applications, thus minimizing undesirable genetic alterations induced by such treatments. In a previous work, the *Rg1 *allele was combined with the small size and short life cycle of MT in the new cultivar Micro-MsK [17]. Here, we developed 'MT-*Rg1*', a near-isogenic line of MT containing the *Rg1 *allele from Micro-MsK. We have also developed a hormone treatment during *Agrobacterium *co-cultivation and regeneration that replaces the use of zeatin, which, despite its elevated cost, is currently adopted in almost all tomato transformation protocols. Combining the high regeneration capacity of 'MT-*Rg1*' and appropriate hormone treatments, we present a simple, inexpensive and efficient method for MT transformation. The genotype and the procedures described here provide an invaluable tool for tomato functional genomics, and will make genetic transformation of MT, and probably other tomato cultivars, more accessible and widespread.

## Results and Discussion

### 'MT-*Rg1*': a near-isogenic line to MT for *in vitro *regeneration improvement

**Figure 1:**
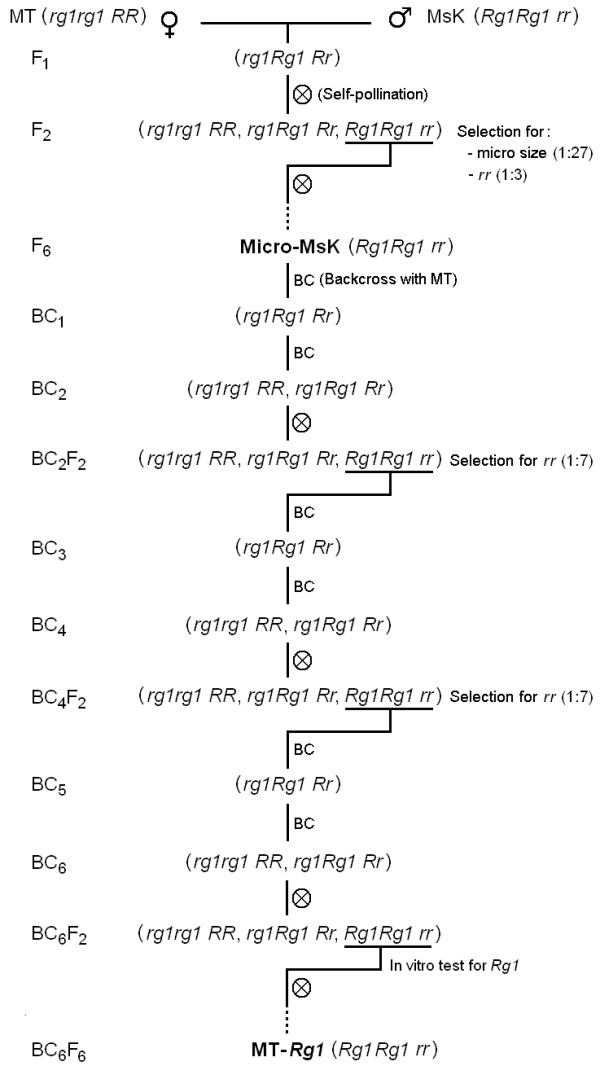
**Introgression of the *Rg1 *allele into the Micro-Tom (MT) background**. The cultivar MsK harbors *Rg1 *linked to the *r *(*yellow flesh*) allele in chromosome 3, which confers yellow color to fruits and was used as a morphological marker. MT was used as pollen receptor in all crosses. In F_2_, recombinants with MT small size and yellow fruits (*rr*) phenotype were selected and allowed to self-pollinate for six generations (F_6_) rendering to the true-type Micro-MsK [17], which was used for further backcrosses (BC) with MT. After every two backcrosses (BC), plants were allowed to self-pollinate rendering to BC_2_F_2_, BC_4_F_2 _and BC_6_F_2_, which were used to select *Rg1 *based on the yellow fruit trait. Seeds from *rr *BC_6_F_2 _plants (BC_6_F_3 _seeds) were germinated in vitro in order to confirm their high shoot formation phenotype from root or cotyledon explants, indicating the presence of *Rg1*. After six generations of self-pollination (BC_6_F_6_) the true-type genotype harboring the morphological marker *r *and the high regeneration allele *Rg1 *was named MT-*Rg1*

**Figure 2:**
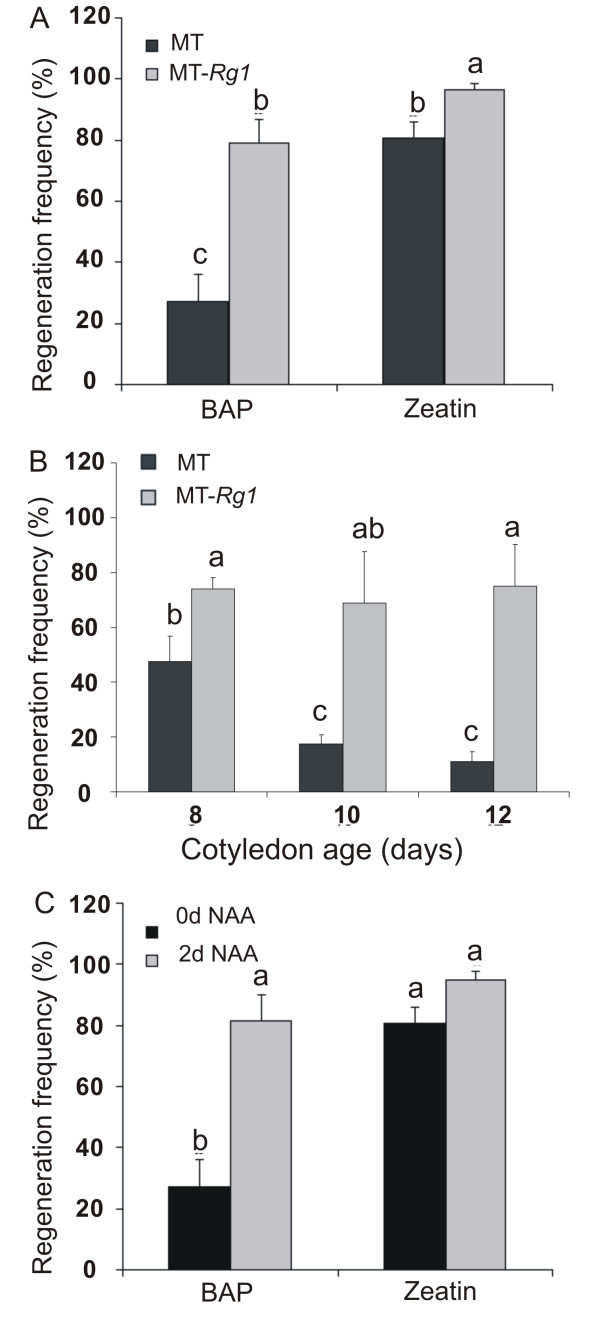
**Shoot regeneration frequency from cotyledon explants in Micro-Tom (MT) and MT-*Rg1***. A. MT and MT-*Rg1 *explants (8 days old) were tested in MS medium supplemented with 5 μM 6-benzylaminopurine (BAP) or 5 μM Zeatin. B. MT and MT-*Rg1 *explants with different ages (days after sowing) were tested in MS medium supplemented with 5 μM BAP. C. MT explants (8 days old) were tested in MS medium supplemented with 5 μM BAP or 5 μM Zeatin with or without 2 days pre-incubation in MS medium supplemented with 0.4 μM 1-naphthaleneacetic acid (NAA). In all treatments, the regeneration frequency (percentage of explants with well formed adventitious shoots) was observed 21 days after explants inoculation. Vertical bars indicate ± standard deviation of the mean (n = 6 Petri dishes with 20 explants each). Different letters indicate significant differences at P ≤ 0.05 (Student's t-test).

The Micro-MsK cultivar, that combines the small plant size of MT with the high *in vitro *regeneration driven by the *Rg1 *allele from *S. peruvianum *[15], was previously described [17]. In the present work, 'Micro-MsK' F_6 _plants were backcrossed (BC) to MT until the sixth generation (BC_6_). After additional six generations of self-pollination (BC_6_F_6_; Figure 1), true-to-type plants harboring the *Rg1 *allele, confirmed by in vitro regeneration tests (Figure 2), were named 'MT-*Rg1*'. The near isogenic genotype exhibits yellow fruits due to the presence of the *r *allele, whose locus is tightly linked to that of *Rg1 *[16]; highly-branched shoots; and delayed leaf senescence, as observed in 'Micro-MsK' [17]. However, 'MT-*Rg1*' plants are slightly shorter than 'Micro-MsK' (data not shown), since 'MT-*Rg1*' is genetically closer to MT.

Besides the potential usefulness of MT-*Rg1 *to improve tomato in vitro regeneration, it could also be used to complement studies on the genetic and physiological basis of morphogenesis [14-16], since it is now fully directly comparable to a well-characterized genetic background (Micro-Tom). Upon request, seeds of 'MT-*Rg1*' are available for interested researchers.

### Improving Micro-Tom in vitro shoot regeneration using the *Rg1 *allele

We compared the regeneration frequency from cotyledon explants of MT and MT-*Rg1 *genotypes, using MS media supplemented with the cytokinin 6-benzylaminopurine (BAP) or zeatin. The regeneration frequency was significantly higher in MT-*Rg1 *in both treatments (Figure 2A), being 2.9-fold and 1.2-fold higher in BAP and zeatin treatments, respectively in comparison to MT. These results confirmed that the *Rg1 *allele increases the regeneration capacity, not only from root explants [16], but also from cotyledonary explants, which is the organ of choice for MT and other tomato varieties transformation [2,3,9]. Despite the fact that zeatin is more expensive and less chemically stable than BAP [18], it is currently adopted for shoot regeneration in most tomato transformation protocols. The reason for zeatin preference in tomato transformation protocols becomes evident when one compares the rate of shoot formation from MT explants onto BAP-containing media against that obtained from zeatin-containing media (Figure 2A). However, regeneration frequency of 'MT-*Rg1*' in BAP was equivalent to that of MT in zeatin (Figure 2A). Therefore MT-*Rg1 *opens the possibility of using BAP instead of zeatin in tomato transformation protocols.

In most tomato transformation protocols, it is recommended the use 8-day-old cotyledons [2,3,9]. Indeed, the regeneration capacity of MT drops dramatically with the increase of the age of cotyledons (Figure 2B). However, the MT-*Rg1 *genotype is able to maintain its high regeneration ability even from fully expanded 12-day-old cotyledons (Figure 2B). This additional advantage of 'MT-*Rg1*' could be useful in the development of more robust (*i.e*. less dependent of restrictive conditions) protocols for tomato transformation.

### Improving Micro-Tom in vitro shoot regeneration in the absence of the *Rg1 *allele

Since the original work of Skoog and Miller in 1950 s, it is believed that induction of specific organs in vitro is manly controlled by the auxin-to-cytokinin ratio, which works not only exogenously [19], but also endogenously [20]. Valvekens et al. [21] developed an Arabidopsis transformation protocol using two steps. The first step was based on a medium that contains a high auxin-to-cytokinin ratio, which induces cell proliferation, denominated Callus-Inducing Medium (CIM). Subsequently, explants were transferred onto a Shoot-Inducing Medium (SIM) with high cytokinin-to-auxin ratio [21]. It is believed that the initial auxin pulse in CIM leads the cells to acquire competence to form shoots in SIM [22,23]. The importance of auxin in the process of acquisition of competence is also evidenced in other developmental processes, such as somatic embryogenesis [24].

Using the same approach employed by Christianson and Warnick [22], we determined that the period for acquisition of competence in MT corresponds to the first two days after explanting and culture in vitro (Lombardi-Crestana et al., unpublished results). An initial pulse of auxin achieved by pre-incubation of cotyledonary explants onto media containing 0.4 μM 1-naphthaleneacetic acid (NAA) for two days, followed by transfer to 5.0 μM BAP media, resulted in a higher regeneration frequency of MT (Figure 2C), compared to BAP without NAA pre-incubation. Thus, combining pre-incubation on NAA followed by incubation on BAP, which are relatively affordable hormones, a regeneration frequency of 80% was attained for MT, similar to values observed for direct incubation onto zeatin media (Figure 2C). The pre-incubation on NAA media, followed by incubation on zeatin also increased in vitro regeneration, but was not statistically different from the results from direct incubation on zeatin (Figure 2C).

It is likely that the improvement in MT regeneration after 2 days pre-incubation on media with 0.4 μM NAA (Figure 2C) is due to the enhancement of competence in an auxin-rich medium. As in the two-step method used by Valvekens et al. [21], we also subsequently transferred the explants to a SIM, which can be supplemented with 5.0 μM BAP or zeatin for tomato [2,3,16,17,25]. However, since excess callus proliferation is negatively correlated with shoot regeneration in tomato [26], a pre-incubation on a Root-Inducing Medium (RIM) containing 0.4 μM NAA, instead of a CIM for tomato [27] may explain the success obtained here. Accordingly, despite the fact that auxin treatment has been long used in tomato regeneration media [25], there is a lack of protocols that consider the need to restrict auxin pulses to the phase of acquisition of competence and to use hormone concentrations that avoid callus formation [9].

Interestingly, the pre-incubation of MT on NAA medium improved shoot regeneration at the same magnitude of that observed using the *Rg1 *allele (Figure 2A and 2C). This reinforces early hypothesis that *RG1 *may be somewhat related to auxin sensitivity or metabolism [28], and suggests that it might be a gene controlling the phase of acquisition of competence in tomato. This assumption is also corroborated by the fact that *Rg1 *not only improves the regeneration of explants, but also extend their responsiveness to an inducing medium (Figure 2B).

### The *Rg1 *allele did not affect *Agrobacterium *infection

**Figure 3:**
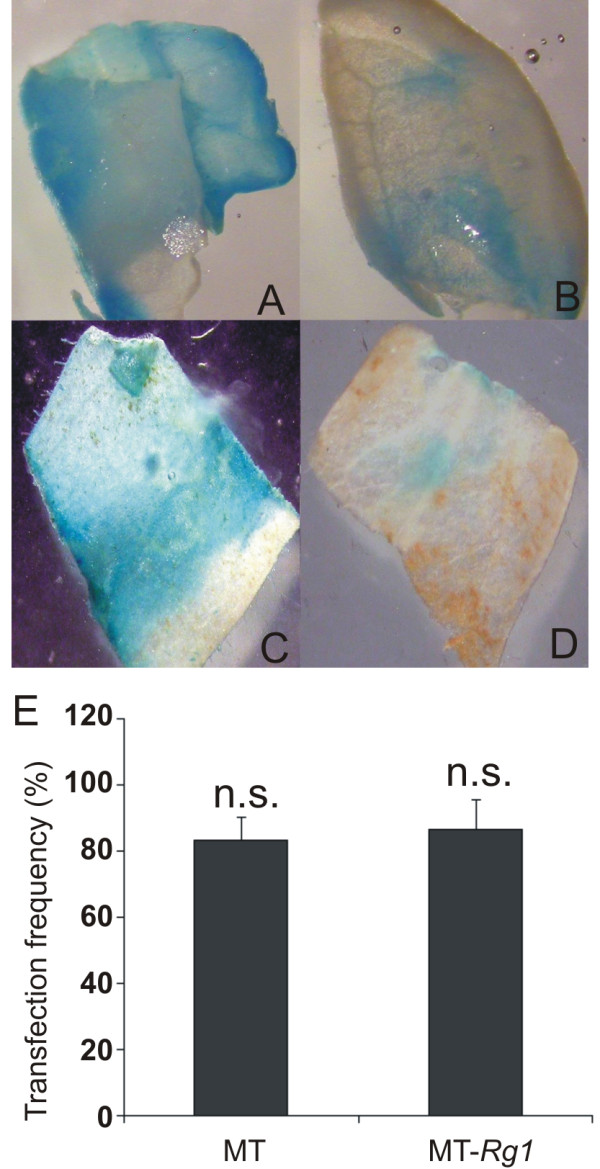
**Transient expression assays**. Cotyledon explants from MT (A, B) and MT-*Rg1 *(C, D) were inoculated with *Agrobacterium tumefaciens *EHA105 harboring the *gusA *reporter gene (see Methods). For the transfection frequency (E) calculation, only explants showing coverage of more than 50% of the surface with GUS staining (A, C) were considered positive. Vertical bars indicate ± standard deviation of the mean (n = 30 explants). The absence of statistic significance (P ≤ 0.05, Student's t-test) is represented by n.s.

To further investigate whether the *Rg1 *allele has any positive or negative pleiotropic effect on *Agrobacterium *infection, and thus affecting genetic transformation, we assayed for transient expression rates using the *A. tumefaciens *strain EHA105, carrying a translational fusion between β-glucuronidase (GUS) and green fluorescent protein (GFP) reporter genes on 'MT-*Rg1*' and 'MT'. *In situ *detection of GUS staining is considered as an indirect measurement of transgene delivery to plant cells [29,30]. Our results showed that gene delivery via *Agrobacterium *was similar between MT and MT-*Rg1 *explants (Figure 3), suggesting that 'MT-*Rg1*' is amenable for genetic transformation using *Agrobacterium*, as MT has already proved to be [1-3].

### The use of MT-*Rg1 *and NAA pre-incubation resulted in a simple, inexpensive and efficient transformation protocol

**Figure 4:**
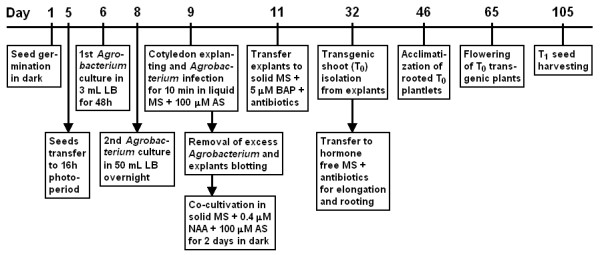
**Schematic representation of the protocol for *Agrobacterium*-mediated transformation of MT**. The horizontal line represents the timeline for each step. Vertical arrows represent successive steps carried out simultaneously

**Figure 5:**
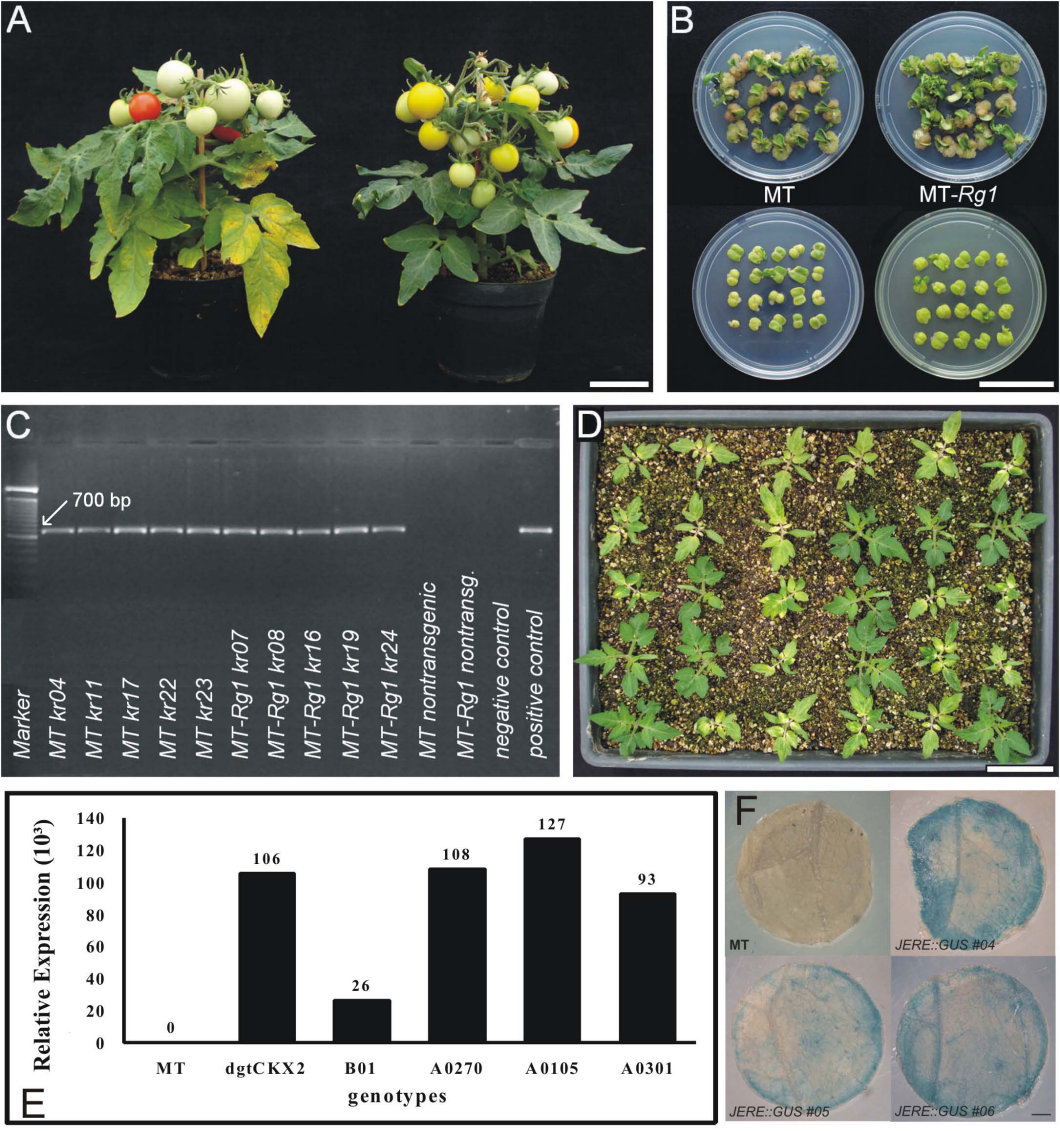
**Phenotype of MT and MT-*Rg1 *and selection of transgenic lines**. A. Phenotype of adult MT and MT-*Rg1 *plants. The presence of the *r *and *Rg1 *alleles resulted in yellow fruits and branched shoot, respectively, in the MT-*Rg1 *genotype (right). B. *In vitro *regeneration in absence (top) and presence (bottom) of 100 mg/L kanamycin. The two plates at the bottom of the figure contain explants pre-incubated with *Agrobacterium *harboring the *nptII*-containing vector pROKII. C. Analyses of *nptII *gene in acclimated T_0 _transgenic plants by agarose gel electrophoresis of PCR amplification of a 700 bp fragment of *nptII *gene and DNA molecular marker (100 bp). D. Selection of segregating T_1 _lines performed in the greenhouse by spraying 400 mg/L kanamycin in 14-day-old seedlings for 5 consecutive days. E. Analysis of *nptII *expression by qRT-PCR of T2 single copy homozygous plants transformed with the pROKII vector containing the *nptII *and *AtCKX2 *genes. F. GUS staining of leaf disks excised from acclimated T_0 _plants transformed with the pGPTV-GUS-KAN containing the *gusA *gene under control of the wound inducible JERE element (AGACCGCC). Note the enhanced staining of the disk borders, which correspond to wounded areas. Bars = 4 cm (A, B and D) and 1 mm (F). Non-transformed MT plants were used as controls (E, F).

The protocol used for *Agrobacterium*-mediated transformation is summarized in Figure 4 and detailed in the Materials and Methods. Based on the high regeneration ability of MT-*Rg1 *(Figure 5A and 5B) and the auxin pre-incubation- increase of regeneration, we tested the combined protocol on transformation efficiency of MT and MT-*Rg1*. Since it had been demonstrated that a 4-fold increase in thiamine concentration in MS was able to improve the regeneration of tomato explants in vitro [11], in the present protocol, the MS media was supplemented with B5 vitamins [31], which contain 100-fold higher thiamine concentration than original MS vitamins. In accordance to these observations, B5 vitamin-based medium is often preferred for tomato regeneration [2,13]. Additional to B5 vitamins, the present transformation protocol also used Acetosyringone (AS) and the antibiotic Meropenem. AS is a phenolic compound that stimulates the induction of *Agrobacterium *virulence genes [32], improving the transformation efficiency of certain species [11,33,34]. The antibiotic Meropenem [35,36] can suppress growth of the bacteria at low concentration (25 mg L^-1^), contrasting to the commonly used antibiotics Cefotaxime and Augmentin, which are utilized at concentrations between 200 and 400 mg L^-1^.

The transformation frequency was higher for MT incubated on NAA pre-treatment followed by cultivation on BAP media, when compared to MT incubated on media containing zeatin (Table 1). MT-*Rg1 *exhibited transformation frequency of 40-80% higher than that observed for MT (Table 1). The transformation frequencies obtained here are comparable to those reported in the best protocols published for tomato transformation [2,3,10,11]. Besides, using BAP instead of zeatin makes transformation more affordable, since zeatin is approximately 100-fold more expensive than BAP. It can be assumed that most of the steps performed in tomato transformation protocols are actually to overcome the low regeneration efficiency of the species. Thus, the use of high regenerating MT-*Rg1 *made the present protocol feasible and reproducible. Furthermore, the present protocol could eventually be extended to other tomato cultivars.

**Table 1:** Transformation efficiency (%) of MT and MT-*Rg1 *in two regeneration media

	Genotypes	
	
Hormones	MT	MT-*Rg1*
21 d Zeatin	21.2 ± 2.4	38.0 ± 7.3
2 d NAA + 19 d BAP	25.0 ± 3.2	37.2 ± 8.9

Means followed by the standard errors (SE) correspond to six independent transformation experiments (n = 6).

MT and MT-*Rg1 *transgenic T_0 _plants (Figure 5C) were acclimatized in approximately 45 days after transformation of explants, and produced T_1 _seeds, that were harvested in a total of 105 days (Figure 4). This rapid production of T_1 _seeds enables further selection of single copy T-DNA insertion based on the segregation of kanamycin resistance in greenhouse (Figure 5D). As evidenced by qRT-PCR and GUS assays (Figure 5E and 5F), the expression level of transgenes can vary widely among independent transformation events. Regardless the causes of the variation in expression among the transgenic lines (*e.g*. the number of copies of T-DNA or the site of insertion), it highlights the importance of a method that increases the efficiency of production of transformants for further selection of those with the best level of gene expression.

The fact that *Rg1 *is genetically linked to the *r *allele (Figure 5A) will not limit the use of MT-*Rg1 *in studies of regeneration and transformation, since when either one or both alleles (the *r *allele or the *Rg1*) would not be desirable, they can be segregated out from the T_1 _generation. To accomplish this, the initial selection can be done using kanamycin application in the greenhouse (Figure 5D), followed by selection for red fruits in the resulting kanamycin-resistant plants. Thus, plants without *r *and *Rg1 *alleles, but containing the transgene, can be selected and multiplied. Moreover, except for studies of carotenoid biosynthesis, the yellow fruit phenotype should not interfere in the interpretation of the results.

### Prospects for tomato functional genomics

The International Solanaceae Genome Project (SOL) started in 2003 with the cooperation of researchers around the world to sequence the 12 tomato chromosomes [37]. Functional genomics tools are increasingly required to explore the information provided by genome projects. MT is considered a useful genotype for functional genomics, and it has already been used for large scale chemical [4,5]; physical [5,38] and insertional [6,39] mutagenesis, as well as for diverse gene expression analysis [2,3,12,13]. Among the advantages of understanding the tomato genome are included the usefulness of this species to address processes difficult or impossible to be investigated in Arabidopsis, such as flowering in a sympodial photoperiod-independent plant; development of fleshy climacteric fruits; interactions of plants with mycorrhiza; and with agronomical relevant pathogens and insects. In order to dissect and study the function of genes regulating the aforementioned processes, a great number of transgenic lines are necessary. This will demand an efficient, simple, and easily reproducible transformation protocol based on inexpensive chemicals, such as the one presented here.

## Conclusions

We generated a new tomato genotype, MT-*Rg1*, with high in vitro regeneration ability. Further, we established that a two-day pre-incubation period of MT cotyledon explants on an auxin-rich medium, sufficient for root induction but not for callus proliferation, also improved MT regeneration. Both strategies enhanced the transformation efficiency of the model system MT, and allowed the use of more affordable hormones for this purposes. The genetic resource and the protocol presented here represent invaluable tools for routine gene expression manipulation and high throughput functional genomics by insertional mutagenesis in tomato.

## Methods

### Plant material, breeding and cultivation

The *Solanum lycopersicum *cultivar Micro-MsK [17], which harbors the dwarfing genes of Micro-Tom (MT) plus the *Rg1 *allele from *S. peruvianum *[16] was crossed and backcrossed to MT by conventional means to obtain a near-isogenic line (Figure 1), named MT-*Rg1*. General-purpose growth of plants was carried out in a greenhouse under automatic irrigation (four times a day), at an average mean temperature of 28°C; 11.5 h/13 h (winter/summer) photoperiod, and 250-350 μmol m^-2 ^s^-1 ^PAR irradiance [natural radiation reduced with a reflecting mesh (Aluminet - Polysack Industrias Ltda, Itápolis, Brazil)]. The miniature plants were grown in 150-ml pots containing a 1:1 mixture of commercial substrate (Plantmax HT, Eucatex, São Paulo; Brazil) and expanded vermiculite, supplemented with 1 g NPK 10:10:10 L^-1 ^substrate and 4 g dolomite limestone (MgCO_3_+CaCO_3_) L^-1 ^substrate. At flowering stage (about 35 days from sowing) plants were supplemented with NPK (*circa *0.2 g/pot). About 40 days after each crossing, mature fruits were harvested and the seed pulp was removed by fermentation for 12-h using commercial baker's yeast (*Saccharomyces cerevisae*, Fermix, São Paulo; Brazil). Seeds were subsequently washed and air-dried.

### Hormone stock solutions

To prepare stock solutions, hormone salts were dissolved by drops of 1 M HCl (BAP and zeatin) or 1 M KOH (NAA), before adding distillated water. All hormone stocks were filter-sterilized (0.2 μm) before adding to sterile media. The pH of the hormone stock solutions was not adjusted and they were added, under sterile conditions, after pH adjustment and autoclaving of the medium. No alterations in the pH of the final medium were observed upon addition of hormone stock solutions. Acetosyringone (AS) stock solution was made in 70% ethanol and added to the medium after filtration (0.2 μm). The concentration of stock solutions used was 5 mM BAP (0.0548 g in 50 mL water); 5 mM zeatin (0.0563 g in 50 mL water), 0.4 mM NAA (0.0037 g in 50 mL water); and 100 mM AS (0.9811 g in 70% ethanol). Using these 1000× stock solutions requires 1 mL of stock per L of final medium.

### Vectors

The plasmid pROKII ([40]; kindly provided by Thomas Schmülling, Free University of Berlin) was used to develop the MT transformation system using *Agrobacterium tumefaciens*. The pROKII contains a *neomycin phosphotransferase II *(*nptII*) gene, which confers resistance to kanamycin, driven by the *nos *promoter. The plasmids pROKII, harboring a cytokinin oxidase gene (*AtCKX2*) driven by the *CaMV 35 S *promoter [41], and the pGPTV-GUS-KAN, containing the *gusA *gene under control of the wound-inducible JERE element (AGACCGCC) [42], were also used for stable transformation. For transient transformation assays, the binary vector pCambia1304, containing a translational fusion between the reporter genes *gusA *and *Egfp5 *and the *hpt *gene that confers hygromycin resistance, both driven by the *CaMV 35 S *promoter [43], was used. The plasmids were introduced into *A. tumefaciens *EHA105 by electroporation.

### Agrobacterium preparation

*Agrobacterium *was initially grown in solid LB medium containing 50 mg L^-1 ^rifampicin (Aventis Pharma Ltda, Suzano, Brazil) and 100 mg L^-1 ^kanamycin (Gibco, Grand Island, NY, USA) for 48 h at 28°C. A single colony was transferred to 3 mL of liquid LB medium supplemented with the rifampicin and kanamycin, as above, and cultivated at 28°C for 48 h at 120 rpm. From this culture, 500 μL were taken and added to 50 mL of fresh LB medium with the same antibiotics. The *Agrobacterium *culture was incubated overnight at 120 rpm and afterwards it was centrifuged at 2000 *g *for 15 min. The pellet was resuspended in liquid basal MS [44], supplemented with 30 g L^-1 ^sucrose and B5 vitamins [31], to an OD_600 nm _of 0.2-0.3. Ten minutes before inoculation, sterile AS (Acros Organics, Morris Plains, NJ, USA) was added to the bacterial suspension to a final concentration of 100 μM.

### Explant inoculation and in vitro regeneration

Seeds from MT and MT-*Rg1 *were surface-sterilized by shaking in 100 mL of 30% (v/v) commercial bleach (2.7% sodium hypochloride) plus two drops of commercial detergent, for 15 min, followed by three rinses with sterile water. The seeds were germinated on half strength MS salts; half strength B5 vitamins; 15 g L^-1 ^sucrose and 6 g L^-1 ^agar (Merck, Darmstadt, Germany). MS original vitamins [44] were also tested for seed germination and explant regeneration. The media pH was adjusted to 5.8 before autoclaving. Approximately 40 seeds were sown per flask containing 30 mL of media. Cultures were sealed with PVC. Cultures were incubated at 25 ± 1°C in the dark for 4 d, followed by 4 d under 16-h photoperiod provided by a 40 W cool white fluorescent tube (c.a. 45 μmol PAR m^-2 ^s^-1^).

Cotyledons were isolated from 8-day-old seedlings. The distal and proximal tips were removed, and the cotyledons were divided transversally in two or three pieces. Explants were placed with the abaxial side down immediately after isolation, with 20 explants per Petri dish (90 × 15 mm), using 6 plates per treatment. Explants were placed onto solid Root Inducer Medium (RIM), composed by MS salts, with B5 vitamins, 30 g L^-1 ^sucrose, 6 g L^-1 ^agar, 0.4 μM NAA (Sigma, St Louis, USA), and 100 μM AS. During explanting, a Petri dish containing potassium permanganate salts was kept inside the laminar flow hood to avoid ethylene accumulation, which can reduce tomato regeneration afterwards [28].

Two drops of Agrobacterium suspension in liquid MS were applied per explants using a micropipette. This amount of suspension was sufficient to cover the explants surface completely, including the borders. Plates were incubated at room temperature, and after 10 min the excess of bacterial suspension was removed with a sterile pipette, and explants were blotted dry on sterile filter paper. Plates were maintained under dark conditions at 28°C for 2 d for co-cultivation. Explants were then transferred to Shoot Inducing Medium (SIM), composed by MS medium with B5 vitamins, 30 g L^-1 ^sucrose, 6 g L^-1 ^agar, 5 μM BAP (Sigma, St Louis, USA) or zeatin (Duchefa, Haarlem, The Netherlands), supplemented with 100 mg L^-1 ^kanamycin and 25 mg L^-1 ^Meropenem (Antibióticos do Brasil Ltda, Cosmópolis, Brazil), and cultivated under 16 h photoperiod at 25 ± 1°C for 3 weeks. One subculture was performed during this period. In all steps, plates were sealed with parafilm. Well-developed shoots (2-4 mm) were separated from the explants and transferred to flasks containing 30 mL hormone-free MS media supplemented with 100 mg L^-1 ^kanamycin and 25 mg L^-1 ^Meropenem to elongate and root for two weeks. Flasks were sealed with PVC. Kanamycyn was used for *in vitro *selection of transgenic lines. To suppress *Agrobacterium *growth over the explants after co-cultivation, besides Meropenem (25 mg L^-1^), 400 mg L^-1 ^Augmentin (GlaxoSmithKline, UK) or 400 mg L^-1 ^cefotaxime (Novafarma, Brazil) were also tested.

### Acclimatization and selection of transgenic lines

Rooted T_0 _plantlets were transferred to the greenhouse for acclimatization in 150 mL pots containing the same mixture described before. Transgenic plants were allowed to self-pollinate, producing T_1 _seeds, and following generations. T_1 _and T_2 _generations were confirmed for kanamycin-resistance in the greenhouse by spraying the kanamycin (400 mg L^-1^) in 14-day-old (from sowing) seedlings for 3-5 consecutive days [45]. Transgenic lines that remained green after kanamycin sprays were selected. Transgenic T_1 _lines that produced only kanamycin resistant T_2 _seedlings were considered homozygous.

To avoid overestimation of transformation frequency, plants originated from the same area in the explant were considered as derived from non-independent transformation events. Transformation frequency was calculated by dividing the total number of independent transformation events (acclimatized plants) by the total number of inoculated explants.

### Transient and stable GUS expression

To confirm successful transient expression, cotyledonary explants were subjected to histochemical assay to detect GUS activity. The number of blue-colored GUS positive spots was correlated with the transient expression rates. Transient expression was evaluated in 30 explants (3 Petri dishes with 10 explants each; n = 3) per treatment. Explants were considered GUS positive when exhibiting more than 50% of surface covered with blue spots. To test stable GUS expression in *JERE::GUS *plants, leaf disks excised from acclimated T_0 _plants were incubated in water for 12 h and then transferred to staining buffer [80 mM sodium phosphate buffer, pH 7.0; 0.4 mM potassium ferrocyanide; 8 mM EDTA; 0.05% Triton X-100; 0.8 mg/mL 5-bromo-4-chloro-3-indolyl-β-D-glucuronide (X-Gluc); 20% methanol] and incubated at 37°C [46]. The reaction was stopped by adding 70% ethanol after 24 h, allowing the removal of chlorophyll from leaf disks. Results from GUS assays were observed under stereomicroscope (SMZ800, Nikon, Japan).

### Molecular analysis

To identify transformants, genomic DNA was extracted from leaflets of acclimated T_0 _plants, using the method described by Fulton et al. [47], and tested for the presence of the *nptII *gene. Negative (DNA from non-transgenic plants and disarmed bacteria) and positive (purified plasmid) controls were employed. The *nptII *gene (~700 bp) was amplified using specific primers (F: 5'-GAGGCTATTCGGCTATGACTGG-3' and R: 5'-ATCGGG AGCGGCGATACCGTA-3'). Each polymerase chain reaction (20 μL) contained 20 mM Tris-HCl; 50 mM KCl; 3 mM MgCl_2_; 125 μM dNTPs; 1 U *Taq *DNA polymerase; 75 ng plant DNA; and 0.25 μM of each primer. The reaction was submitted to the following conditions: 1 min at 94°C; 30 cycles of amplification (1 min at 94°C; 30 s at 60°C; and 2 min at 72°C) and a final cycle of 10 min at 72°C. The amplified fragments separated in 1% agarose gel electroforesis using 1/2 Tris-borate buffer stained with Ethidium bromide.

For quantitative reverse transcription amplification (qRT-PCR), total RNA extraction from leaflets was carried out using a lithium chloride (LiCl) protocol [48]. Three μg of each total RNA sample, treated previously with DNAseI (Fermentas), were reverse transcribed in a 20-μL reaction using the SuperScript III kit (Invitrogen), according to the manufacturer's recommendations. Quantitative PCR from this cDNA was performed with primers specific for the *nptII *gene (F: 5'-GGCTATGACTGGGCACAACA-3' and R: 5'-GCAGGAGCAAGGTGAGATGAC-3') and for the *GAPDH *(glyceraldehyde 3-phosphate dehydrogenase) gene (F: 5'-TCCATCACAGCCACTCAGAA-3' and R: 5'-TCAACCACGGACACATCAAC-3') as an internal reference. Samples at a concentration 1:10 were evaluated in a RotorGene 3000 thermocycler (Cobertt Research) in triplicate. Cycle threshold (C_T_) values were recorded and amplification efficiencies were determined by a dilution series using the software Rotor-Gene Real-Time Analysis 6.0. Relative expression values were calculated according to Pffafl [49].

## Competing interests

The authors declare that they have no competing interests.

## Authors' contributions

LEP performed transformation assays, acclimatization and selection of transgenic plants and participate in preparation of the manuscript. SLC performed all crosses and selection of genotypes. MSA performed *in vitro *regeneration experiments. DCS and LB performed the molecular analyses. VQ performed transient expression assay and participate in preparation of the manuscript. AF participated in conceiving the project and manuscript preparation. LEPP conceived the project, supervised transformation, regeneration and preparation of the manuscript. All authors read and approved the final manuscript.
